# Cold shock treatment extends shelf life of naturally ripened or ethylene-ripened avocado fruits

**DOI:** 10.1371/journal.pone.0189991

**Published:** 2017-12-18

**Authors:** Jiao Chen, Xixia Liu, Fenfang Li, Yixing Li, Debao Yuan

**Affiliations:** 1 Hainan Key Laboratory of Banana Genetic Improvement, Haikou Experimental Station, Chinese Academy of Tropical Agricultural Sciences, Haikou, China; 2 Hubei Key Laboratory of Edible Wild Plants Conservation and Utilization, Hubei Normal University, Huangshi, China; South China Agricultural University, CHINA

## Abstract

Avocado is an important tropical fruit with high commercial value, but has a relatively short storage life. In this study, the effects of cold shock treatment (CST) on shelf life of naturally ripened and ethylene-ripened avocado fruits were investigated. Fruits were immersed in ice water for 30 min, then subjected to natural or ethylene-induced ripening. Fruit color; firmness; respiration rate; ethylene production; and the activities of polygalacturonase (PG), pectin methylesterase (PME), and endo-β-1,4-glucanase were measured. Immersion in ice water for 30 min effectively delayed ripening-associated processes, including peel discoloration, pulp softening, respiration rate, and ethylene production during shelf life. The delay in fruit softening by CST was associated with decreased PG and endo-β-1,4-glucanase activities, but not PME activity. This method could potentially be a useful postharvest technology to extend shelf life of avocado fruits.

## Introduction

Avocado is an important tropical fruit with high commercial value. The fruit is reported to exhibit some health-promoting properties owing to its high concentration of monounsaturated fatty acids and significant amount of beneficial healthy compounds such as tocopherols (vitamin E), sterols, and folate [1]. About 547,848 ha of avocado were planted in 2014 in about 70 countries, with an annual production of about 5.03 million tons (FAOSTAT); Mexico is the largest producer, followed by Dominican Republic, Peru, Indonesia, Colombia, Kenya, China, the USA, and Rwanda (FAOSTAT).

Avocado is a typical climacteric fruit with a relatively short storage life owing to rapid flesh softening and decay [2–4]. Applying 1-methylcyclopropene seems to be promising for delaying avocado fruit ripening, but increases the risk of decay [5–6]. Cold storage is effective for extending the postharvest life of avocado fruits [5]. However, the fruits are susceptible to chilling injury, which manifests as black skin and pits [7–9]. In some countries with developed avocado industries, such as Peru and South Africa, ‘step-down’ temperature technology is widely applied for fruit export. In this technology, temperatures are gradually decreased by 1−2°C during storage each week, with a final temperature of ≥ 3.5°C [10]. However, in the major avocado production countries, such as Indonesia, Kenya, China, Rwanda, and Brazil, avocados are mainly sent to local markets where more than 30% of the fruits are lost after harvest owing to poor cold storage facilities and postharvest handling systems [11–12]. Therefore, it is necessary to develop alternative, less sophisticated, low-cost technologies that do not require refrigeration to extend the postharvest life of avocado fruits.

Pre-cooling is the first most important handling process in the entire cold supply chain management of fruits [13]. It is usually recommended that avocado fruits should reach the packhouse within two hours after harvest [5]. On arrival, the fruits should be pre-cooled to 16°C or colder [5]. Commercially, hydro-cooling is the most common method used at the packhouse [5]. Recently, cold shock treatment (CST) has attracted extensive attention as a modified pre-cooling approach. CST rapidly lowers the internal temperature of agricultural products by cold air or ice water, which can extend shelf-life and improve the quality of some fruits and vegetables. The beneficial effect of rapid cooling with low-temperature air or water for inhibiting chilling injury development in West Indian avocados was also reported [14]. In a preliminary study, short-term ice water treatment delayed the softening of ‘Hass’ avocado fruits at room temperature. However, there is a need to better understand the effects of CST on ripening processes of harvested avocado fruits.

The objective of this study was to elucidate the effect of CST on physiological attributes of naturally ripened and ethylene-ripened “Hass” avocado fruits at 20°C with respect to shelf life and postharvest quality.

## Materials and methods

### Plant material and treatments

‘Hass’ (*Persea americana* var. guatemalensis) avocados with a dry matter content of 29.3 ± 0.5% were harvested from the North and South fruit market in Haikou, Hainan Province, China. Fruits were transported to the laboratory within 3 h after harvest and then selected for uniform shape, color, and size. In the first experiment, avocado fruits were dipped into cold water (0, 2, or 4°C) for 15, 30, 45, 60, 90, or 120 min. Following cold water treatments, the fruits were removed, stabilized for 3 h at room temperature, and stored at 20°C and 85–90% relative humidity (RH). After 6 d of storage, fruit firmness was evaluated to determine optimal CST conditions. Since immersion with cold water at 0°C for 30 min was the most effective in maintaining fruit firmness, these conditions were used in the subsequent experiments. In the second experiment, the fruits were subjected to the following treatments: 1) natural ripening; 2) natural ripening following CST; 3) ethylene-induced ripening; and 4) ethylene-induced ripening following CST. For ethylene-induced ripening, the avocado fruits were treated with 100 μL L^−1^ ethylene in sealed jars for 24 h at 20°C. Afterward, the fruits were held at 20°C and 85–90% relative humidity (RH) for 5 or 10 d. Samples were collected at 1-d and 2-d intervals for natural ripening and ethylene-induced ripening, respectively. Color, fruit firmness, respiration rate, and ethylene production rate were evaluated immediately. For the enzymatic activity assays, fruit tissue samples were collected, frozen in liquid nitrogen, and stored at -80°C for further analysis. A total of 300 avocado fruits were used in this study. Each group contained three replicates with 25 fruits in each replicate. At any sampling time, each replicate contained 5 fruits.

### Peel color measurement

The peel color was measured at the equatorial region (four readings per fruit), using a Minolta Chromometer CR-200 (Minolta, Tokyo) to evaluate the lightness (L*), chroma value (C*), and hue angle. Four measurements were performed on each fruit and 15 fruits were examined per treatment.

### Fruit firmness

One side of the avocado fruit was peeled with a stainless steel knife (about 1 mm in thickness), and then three measurements were performed by puncture test using a Universal Testing Machine (UTM) TA.XT.PLUS Texture Analyzer (Stable Micro Systems, Godalming, Surrey, UK) equipped with a HDP/90 platform, a SMS P/2 needle probe (a stainless steel cylinder of 2 mm in diameter with a conical needle bit) and a 1 kg load cell. Fruit firmness was defined as the maximum break force (*Fsk*) and expressed in Newtons (N), which corresponded to resistance to the needle probe penetration. Fifteen fruits were examined per treatment.

### Respiration rate and ethylene production rate

Five fruits were sealed inside a 6.4-L glass jar for 2 h at 20°C. Then, 5 mL of headspace was withdrawn and analyzed by gas chromatography, as described by Zhang et al. (2011) [15]. Respiration rate and ethylene production rate were expressed on a fresh weight (FW) basis.

### Enzyme extraction and assays

Frozen pulp tissues were ground using a chilled DFY-500C grinder, and then 2 g of these powders were homogenized with the pre-cooled buffer at 4°C to prepare extract for assays of the following enzymes: 20 mL Tris-HCl (20 mM, pH 7.0) containing cysteine-HCl (20 mM), EDTA (20 mM) and 0.05% Triton X-100 (w/v) for polygalacturonase (PG, EC 3.2.1.15), pectin methyl esterase (PME, EC 3.1.1.11), and cellulase (EC 3.2.1.4). The homogenates were centrifuged at 15,000 × *g* for 30 min at 4°C. The supernatants were collected and used for the enzymatic assays.

PME activity was evaluated according to the method described by Hangermann and Austin (1986) [16] with some modifications. The reaction mixture contained 1 mL of pectin solution (0.01% aqueous solution adjusted to pH 7.5 using 0.1 N NaOH), 0.2 mL of NaCl (0.15 M), 0.1 mL of bromothymol blue solution (0.01%), 0.2 mL of water, and 0.1 mL of enzyme extract. Absorbance was measured at 620 nm immediately after adding the enzyme extract and then gently shaken for 0 (A_0_) or 3 min (A_1_). The difference between A_0_ and A_1_ was used for calculating PME activity. One unit was defined as the amount of the enzyme required to liberate 1 μmol of methyl ester per minute.

PG activity was assayed by the method described by Pathak and Sanwal (1998) [17]. The reaction mixture contained 0.2 mL of sodium acetate (200 mM, pH 4.5), 0.1 mL of NaCl (200 mM), 0.3 mL of polygalacturonic acid (PGA, 1% aqueous solution adjusted to pH 4.5) and 0.1 mL of enzyme extract in a total volume of 1.0 mL. The reaction was initiated by adding the PGA substrate. The mixture was incubated at 37°C for 1 h, followed by adding 3, 5-dinitrosalicylic acid (DNS). The reaction was terminated by heating the reaction mixture in a boiling water bath for 5 min. In the control tube, the substrate was added after heat treatment. The formation of reducing groups was estimated against D-galacturonic acid as the standard, based on the absorbance at 540 nm. PG activities were calculated according to the method described by Awad and Young (1979) [18].

Endo-β-1,4-glucanase activity was measured based on the reduction in viscosity of carboxymethylcellulase (CMC), as described by Durbin and Lewis (1988) [19]. Measurement was initiated by adding 0.2 mL of enzyme extract to 0.4 mL of CMC (1.30% in 20 mM Tris-HCl, pH 7.0) solution and then mixing thoroughly. The initial viscosity was measured by drawing the solution to the zero mark in a 0.1-mL glass pipette, and then suction was released. The drainage time (*T*1) was recorded with a stopwatch. A second reading (*T*2) was taken after 2 hours. Calculations were made according to the method described by Awad and Young (1979) [18].

## Results and discussion

### Optimal CST

**Fig 1:**
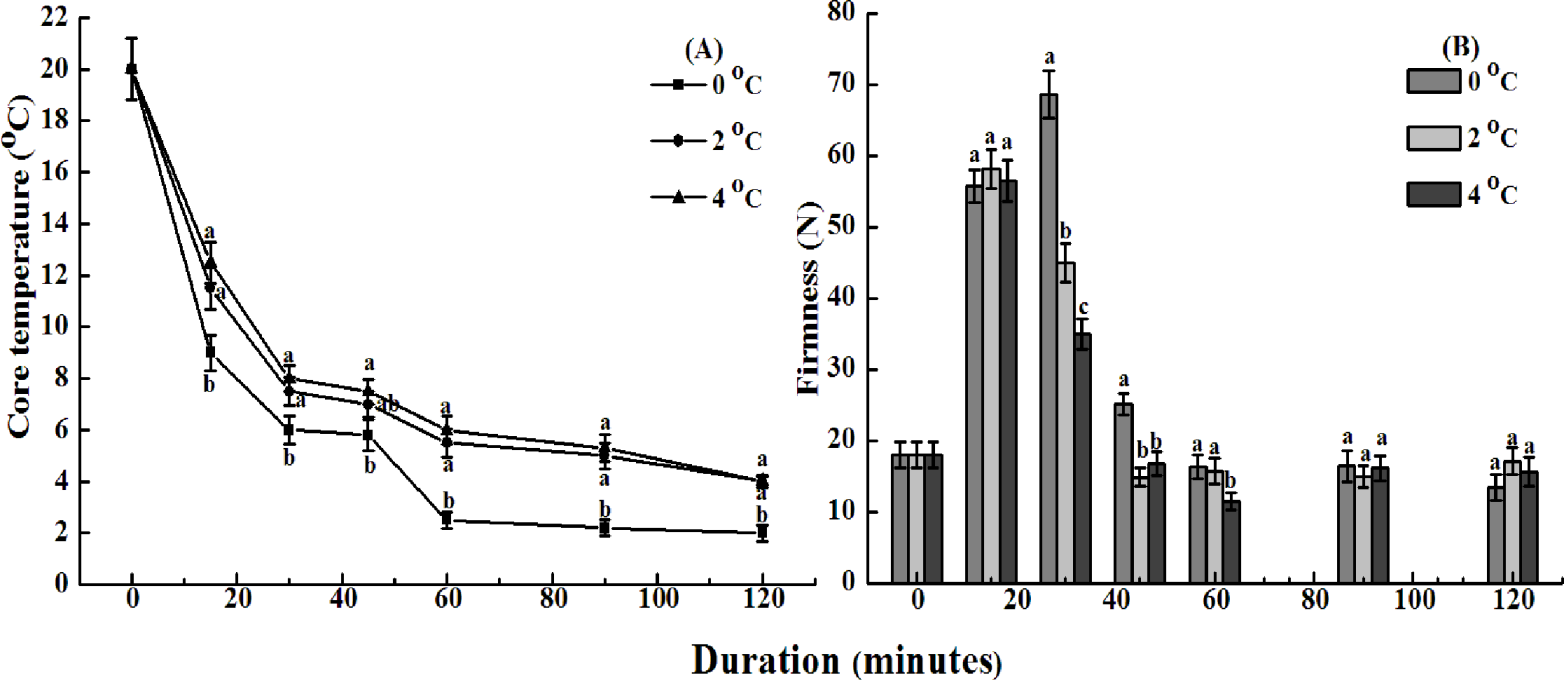
**Changes in core temperature of avocado fruits treated with cold shock at 0, 2 or 4**°**C for 0 to 120 minutes (A) and the corresponding firmness after 6-day storage at 20**°**C and 85–90% RH (B).** Different letters at any storage time indicate significant differences (P<0.05).

The changes in core temperature of avocado fruits treated with CST are shown in Fig 1A. Core temperature declined sharply to 6–8°C within the first 30 min, continued to decline slowly within the next 30 minutes, and thereafter remained almost constant. Dynamic stage and static stage were divided obviously, judging from whether the cooling rates of the tissues were higher or lower than 0.1°C/min [20]. When CST was conducted with cold water at 0, 2, and 4°C, it took about 60, 30, and 29 minutes, respectively, to reach a cooling rate of the center of tissues at the center static cold shock stage (Fig 1A). CST with ice water had obviously better heat transfer efficacy. The core temperature of the avocado fruits declined to 6 and 2.5°C after immersion in ice water for 30 and 60 min, respectively (Fig 1A). A mass-average temperature of 6.7°C could be achieved through CST with ice water for 33 minutes when the initial temperature of avocado fruits was 32.2°C [14].

Symptoms of chilling injury of avocado fruits were not observed in cold shock-treated fruits (even for 120 minutes with ice water) after 6 d of storage at 20°C. Fruit firmness can directly reflect the ripening status and storage life [21]. As shown in Fig 1B, compared with that of control fruit, cold water immersions for 15 or 30 min maintained firmness well, and ice water treatment for 30 min was the most effective. However, CST with longer durations did not inhibit fruit softening. Thus, ice water treatment for 30 min was the optimal CST, and these conditions were used in the subsequent experiments. Previous studies showed that treatment with cold water at 3°C for 40 min or 0°C for 1 h are the optimal CSTs for extending shelf life of cucumber and banana, respectively [21–22]. Therefore, it appears that different types of fruits respond differentially to CST.

### Fruit color

**Fig 2:**
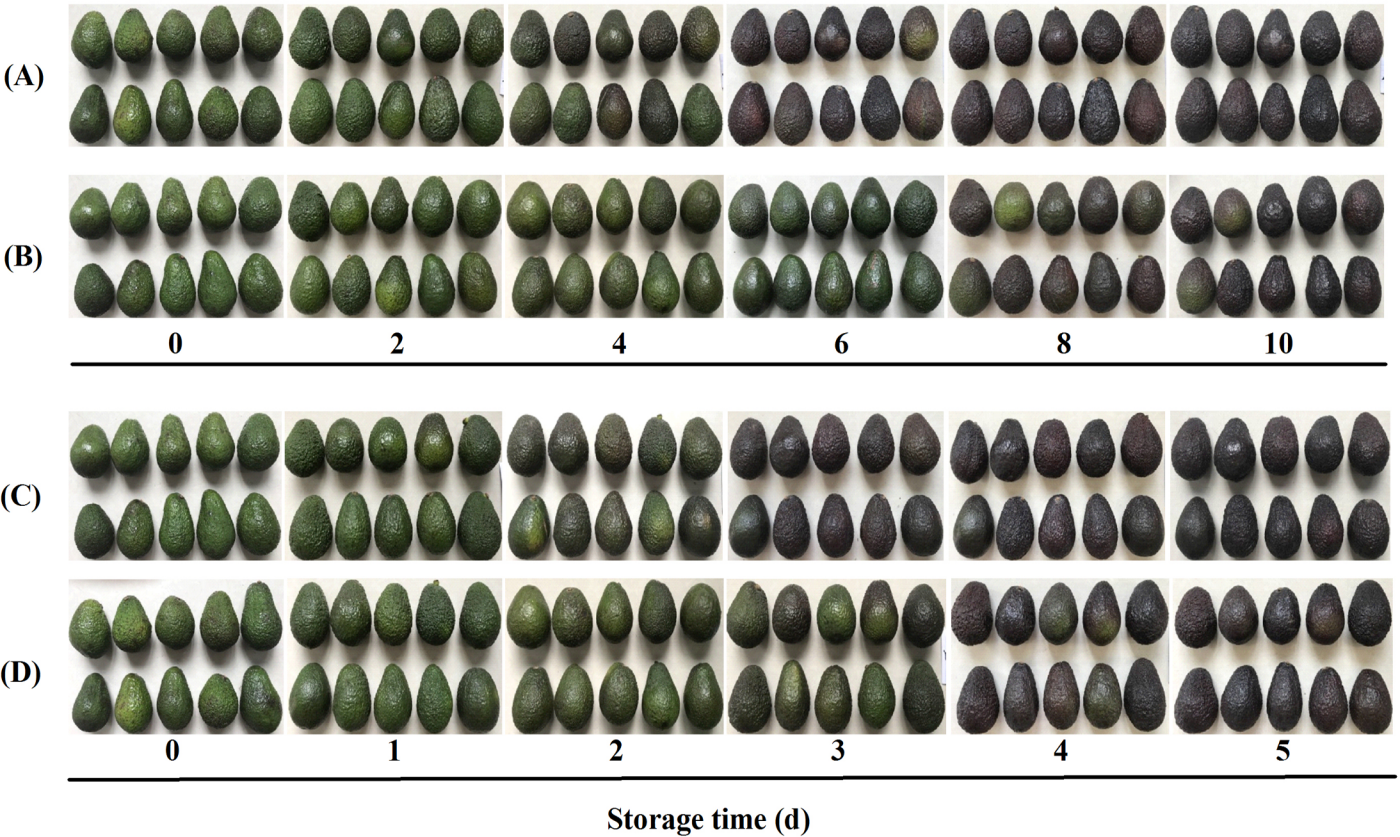
Visual appearance of the avocado fruits. Natural ripening without cold shock treatment (CST) (A); Natural ripening following CST (B); Exogenous ethylene-induced ripening without CST (C); and Exogenous ethylene-induced ripening following CST (D).

Avocado fruit ripening is characterized by fruits turning purplish black. In the present study, control avocado fruits upon natural and ethylene-induced ripening turned purplish black at 4 d and 2 d, respectively. CST markedly delayed peel color change of harvested avocado fruits treated with or without ethylene (Fig 2). Uneven ripening, one of the common problems for ‘Hass’ avocado fruit, was observed in our study. Fruits treated with exogenous ethylene exhibited a lower variability than fruits in other groups, further confirming the commercial advantage of exogenous ethylene application [23–24].

**Fig 3:**
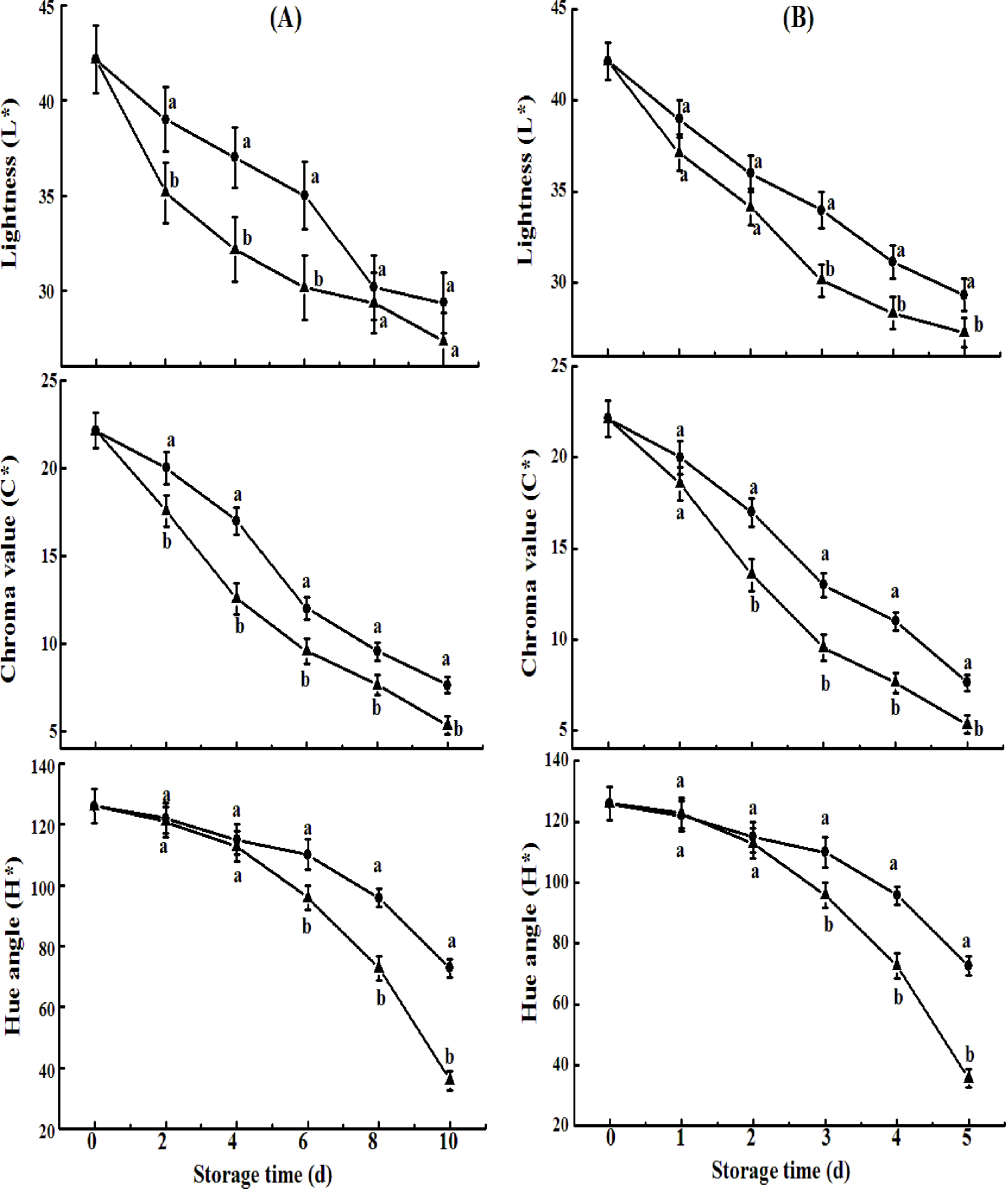
**Skin color (lightness, chroma value, and hue angle) of avocado fruits upon natural (A) ripening or ethylene-induced ripening (B) with (●) or without (▲) cold shock treatment (CST) during 10- or 5-day storage at 20°C and 85–90% RH.** Vertical bars represent the standard error of three replicates. Different letters at any storage time indicate significant differences (P<0.05).

Several color parameters were also measured to evaluate the effect of CST on visual appearance. L* indicates the lightness ranging from black to white, C* represents the color saturation that varies from dull to vivid, and hue angle (h*) refers to a color wheel. As shown in Fig 3, L* values decreased to 27.3 and 27.51 from the initial value of 42.17, indicating that the fruit turned purplish black, while the values for cold shock-treated avocado fruits were 29.35 and 29.86, respectively. Similarly, a less intense decrease in lightness and chroma were observed in cold shock-treated fruits, with or without ethylene exposure. These results indicated that CST maintained the bright green color of harvested avocado fruit well, and might decreas the sensitivity of avocado fruits to ethylene. Overall, CST resulted in 4-d and 1-d prolonged in shelf life of avocado fruits upon natural and ethylene-induced ripening, respectively.

Different color change responses to CST occur depending on the fruit variety. Suppressed color change in cucumber upon CST was reported by Zhao et al. (2017) [25], while improved rind color of ‘Nules Clementine’ mandarin upon CST was observed by Barry and van Wyk (2006) [26]. Partially ripened avocados are often held in cold storage before selling, or avocados without any prior ripening are stored, followed by ethylene treatment and final ripening [27]. Obviously, CST prolonged the green life of ‘Hass’ avocado fruits at room temperature regardless of ethylene treatment in this study.

### Fruit softening and cell wall hydrolases

**Fig 4:**
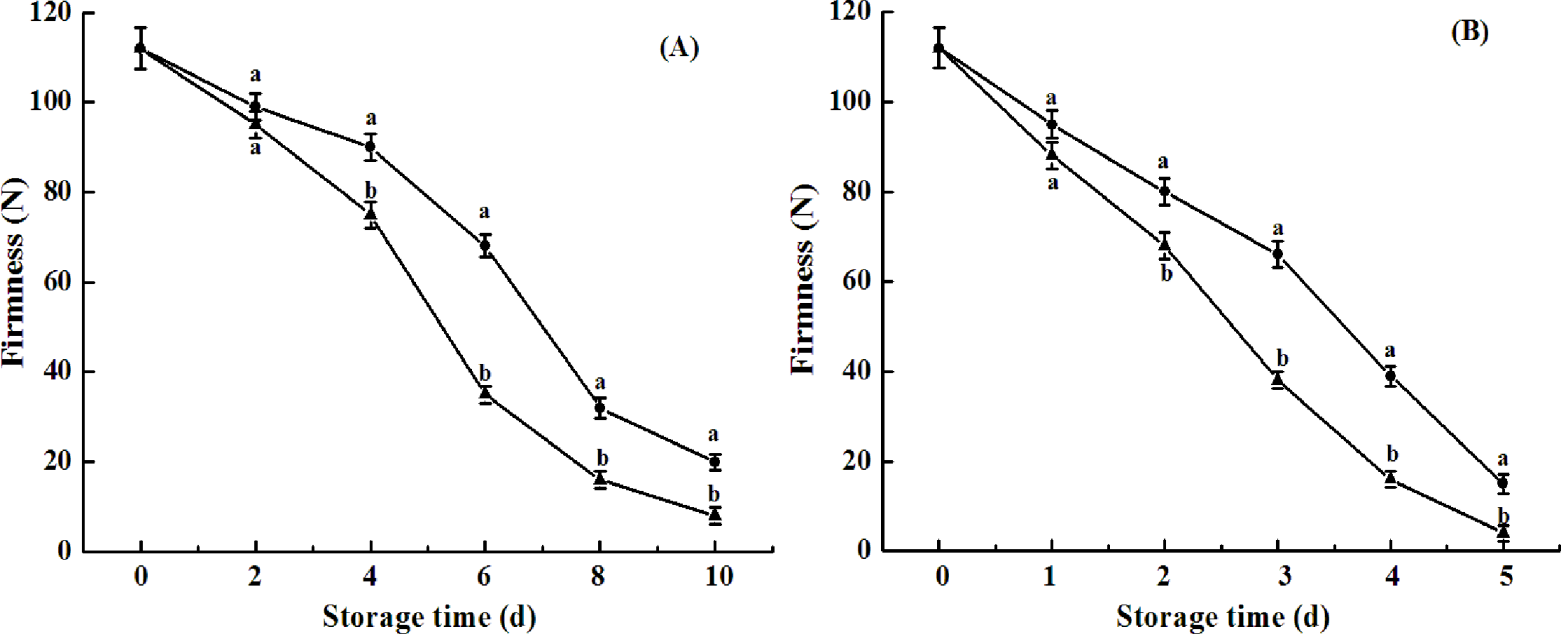
**Firmness of avocado fruits upon natural (A) or ethylene-induced (B) ripening with (●) or without (▲) cold shock treatment (CST) during 10- or 5-day storage at 20°C and 85–90% RH.** Vertical bars represent the standard error of three replicates. Different letters at any storage time indicate significant differences (P<0.05).

Avocado is a typical climacteric fruit. Rapid softening upon ripening is another important characteristic of avocado fruits. As shown in Fig 4, avocado fruit firmness rapidly decreased after harvest (Fig 4). After 8 d and 4 d of shelf life, firmness decreased to 16 N and 15 N from the initial value of 112 N in naturally ripened fruit and ethylene-ripened fruit, respectively. CST significantly suppressed this decrease in firmness during storage (Fig 4). Delayed softening by CST has been also reported in banana, cherry, cucumbers, and carrots [21–22, 25, 28]. Arpaia et al. (2015) [27] pointed out that firmness values close to 19 N for ‘Hass’ avocado fruits could be an indicator of ‘near ripe’ while 4.4–6.7 N was determined as an ‘eating firmness’.

**Fig 5:**
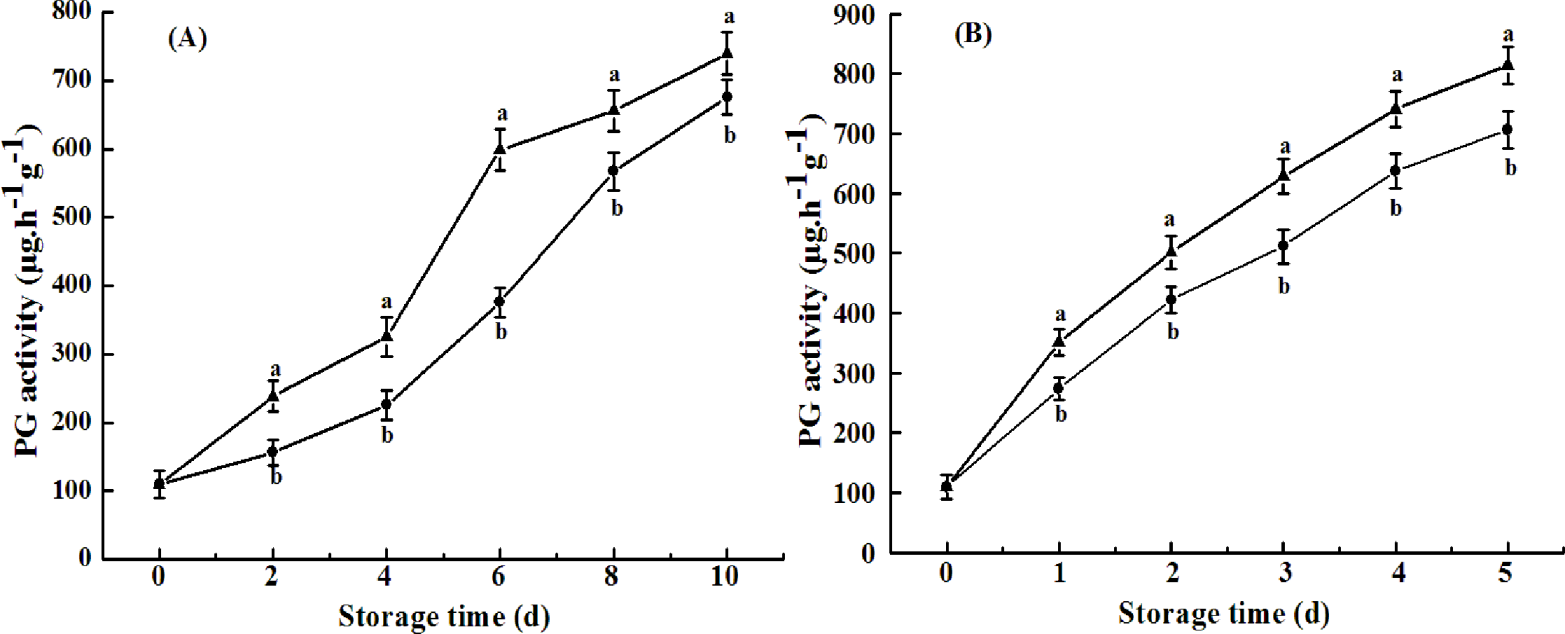
**PG activity of avocado fruits upon natural (A) or ethylene-induced (B) ripening with (●) or without (▲) cold shock treatment (CST) during 10- or 5-day storage at 20°C and 85–90% RH.** Vertical bars represent the standard error of three replicates. Different letters at any storage time indicate significant differences (P<0.05).

**Fig 6:**
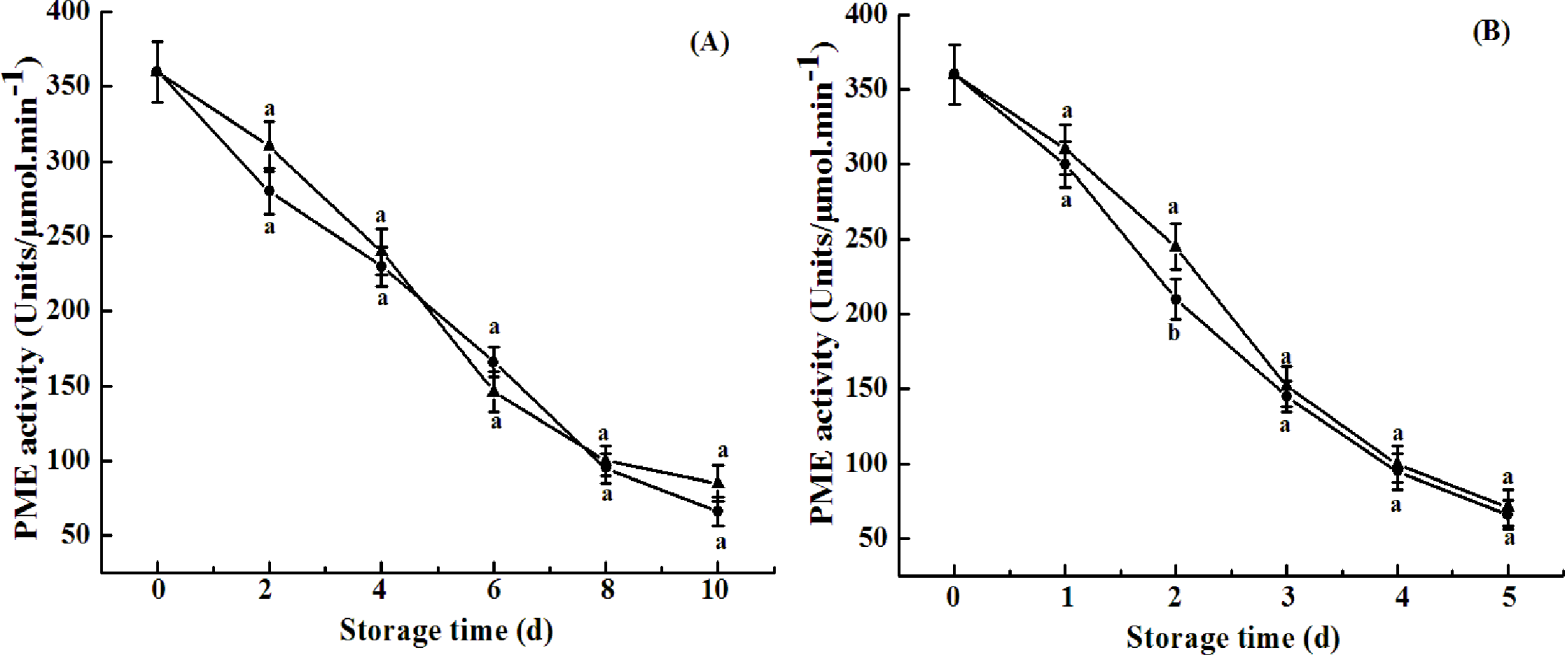
**PME activity of avocado fruits upon natural (A) or ethylene-induced (B) ripening with (●) or without (▲) cold shock treatment (CST) during 10- or 5-day storage at 20°C and 85–90% RH.** Vertical bars represent the standard error of three replicates. Different letters at any storage time indicate significant differences (P<0.05).

Plant cell walls are complex matrices of polysaccharides, mainly consisting of pectin, hemicellulose, and cellulose. Depolymerization of pectin and hemicellulose plays an important role in fruit ripening, leading to the disassembly of cellulose and hemicellulose network and a decrease in fruit firmness [29]. The involvement of polygalacturonase (PG), pectin esterase (PE), and pectate lyase (PL) in the enzymatic degradation of pectin polysaccharides has been well-documented [30]. PG hydrolyzes α-1,4-glycosidic bonds between two galacturonic acid residues, following de-esterification of pectin by PE. In the present study, PG activities in naturally ripened fruit and ethylene-ripened fruit increased from 110.21 to 656 or 741 μg h^-1^g^-1^ 8 d and 4 d after harvest, respectively. However, the activities were 567 and 637 μg h^-1^g^-1^ in cold shock-treated fruits 8 d and 4 d after treatment, respectively. The suppressed PG activities by CST (Fig 5) were concurrent with the delayed fruit softening in both naturally ripened fruit and ethylene-treated fruit (Fig 4), indicating that PG activity is one of the key modulating factors for fruit softening. Unlike PG activity, PME activity of avocado fruits tended to decrease throughout the shelf life, which was consistent with results of a previous study [31]. No significant difference in PME activity between control and cold shock-treated fruits was observed (Fig 6). However, Zhang et al. (2010) [22] reported that activities of PME and PG during banana fruit ripening were suppressed by CST. The result indicated that cell wall degradation-related enzymes in different varieties of fruits respond differentially to CST.

**Fig 7:**
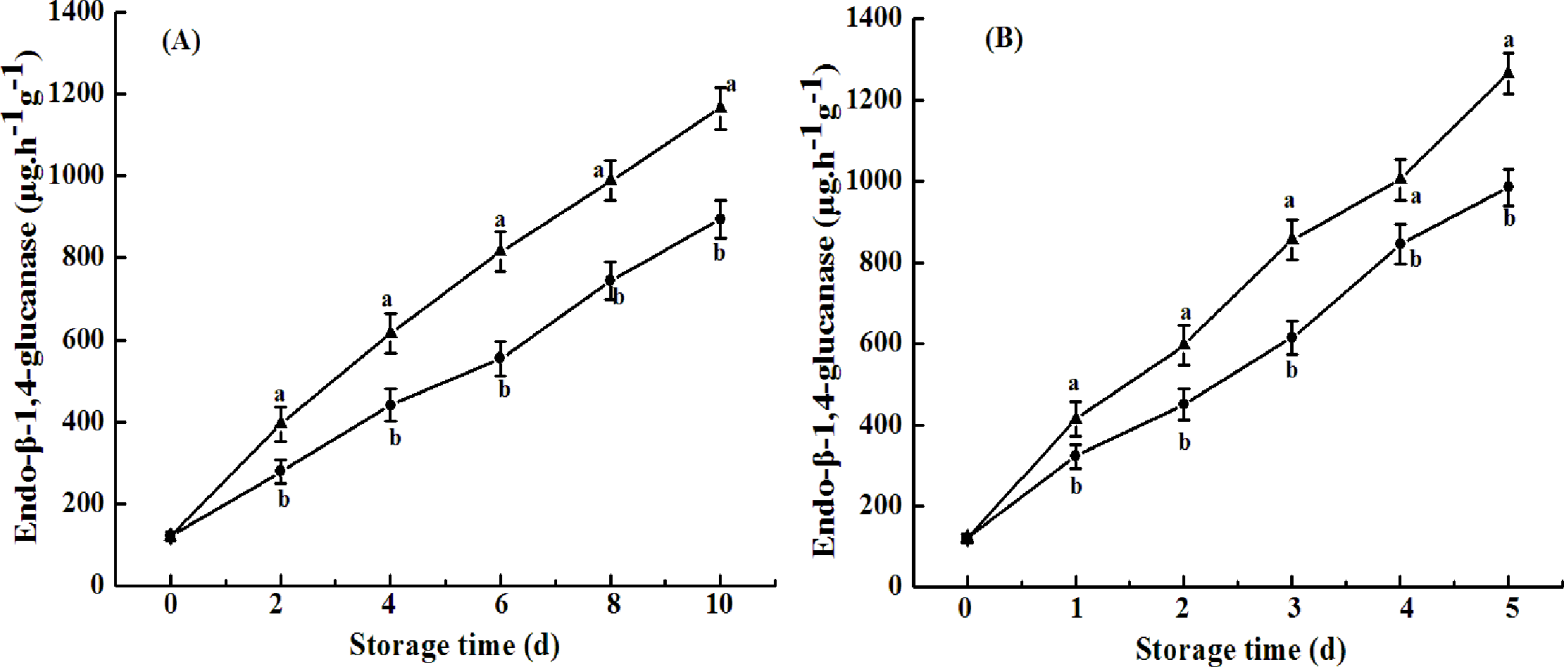
**Endo-β-1,4-glucanase activity of avocado fruits upon natural (A) or ethylene-induced (B) ripening with (●) or without (▲) cold shock treatment (CST) during 10- or 5-day storage at 20°C and 85–90% RH.** Vertical bars represent the standard error of three replicates. Different letters at any storage time indicate significant differences (P<0.05).

In addition, endo-β-1,4-glucanase may also play a role in disassembling the cellulose and hemicellulose network during fruit softening. The endo-β-1,4-glucanases, or cellulases, which are proposed to degrade hemicellulose, are related to fruit softening [32]. In this study, we found that endo-β-1,4-glucanase activity in cold shock-treated avocado fruits was significantly lower than that in control fruit throughout the shelf-life regardless of treatment with ethylene (Fig 7).

Overall, the decreases in activities of PG and endo-β-1, 4-glucanases by CST possibly were beneficial to inhibiting pectin and hemicellulose degradation, consequently, leading to delayed softening of avocado fruits.

### Respiration rate and ethylene production rate

**Fig 8:**
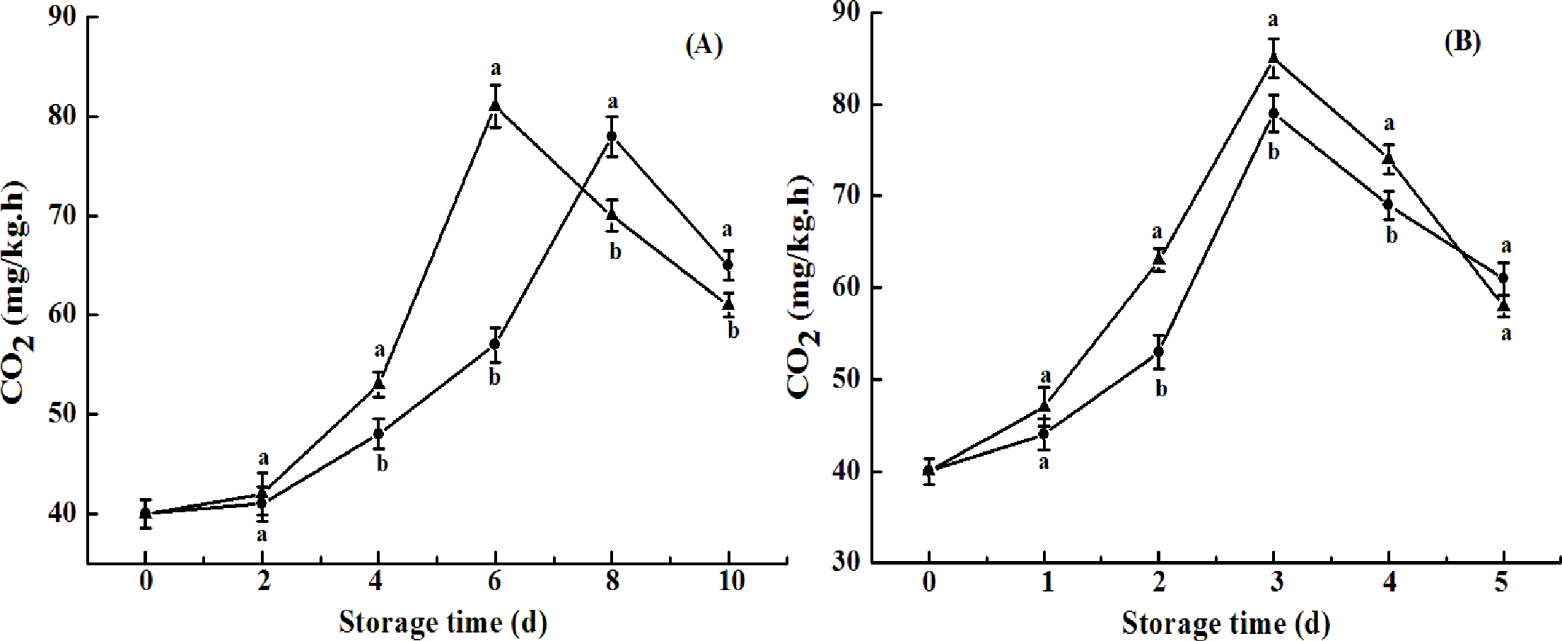
**Respiration rate of avocado fruits upon natural (A) or ethylene-induced (B) ripening with (●) or without (▲) cold shock treatment (CST) during 10- or 5-day storage at 20°C and 85–90% RH.** Vertical bars represent the standard error of three replicates. Different letters at any storage time indicate significant differences (P<0.05).

**Fig 9:**
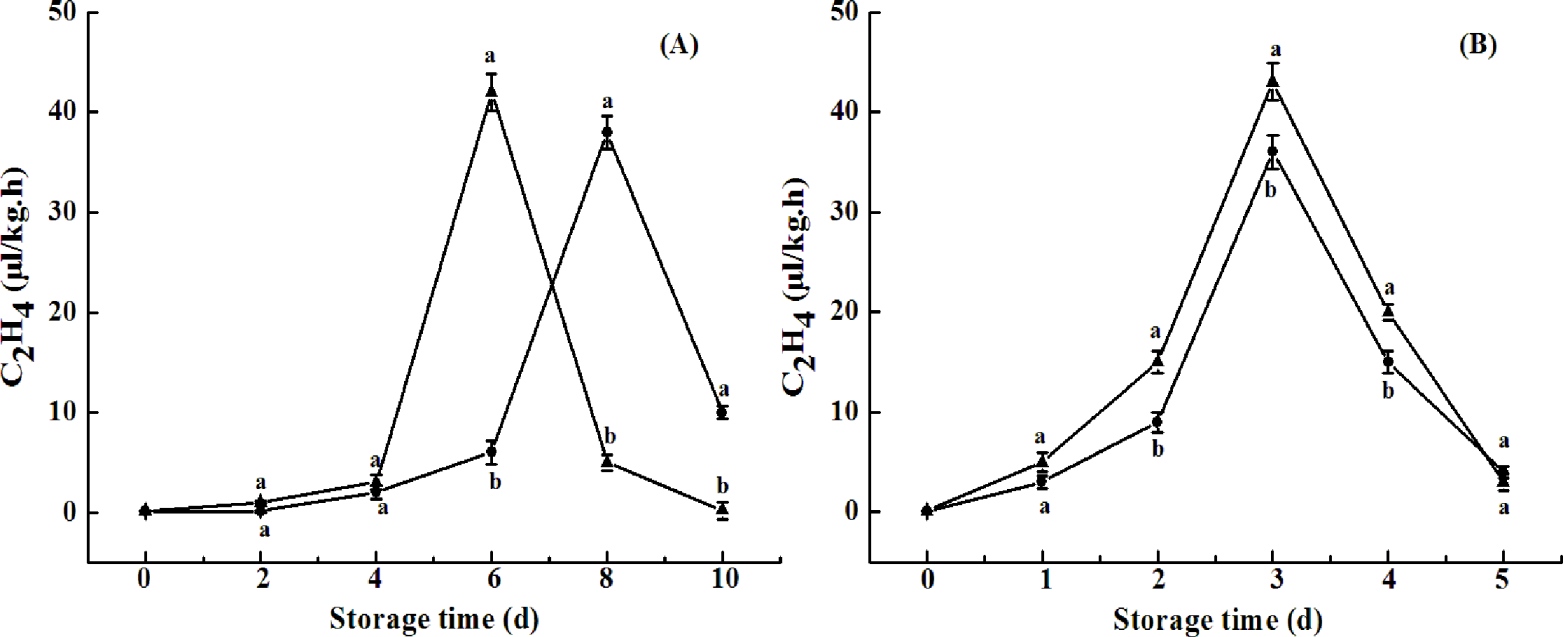
**Ethylene production rate of avocado fruits upon natural (A) or ethylene-induced (B) ripening with (●) or without (▲) cold shock treatment (CST) during 10- or 5-day storage at 20°C and 85–90% RH.** Vertical bars represent the standard error of three replicates. Different letters at any storage time indicate significant differences (P<0.05).

Changes in respiration rate and ethylene production rate are shown in Figs 8 and 9. The respiration rate of the naturally ripened control fruit peaked at day 6, with a value of 81 mg kg^−1^h^−1^ (Fig 8A). CST delayed the peak of respiration rate (Fig 8A). Furthermore, a lower respiration rate of the fruit was observed upon exogenous ethylene-induced ripening with CST (Fig 8B). Compared with those for respiration rate, ethylene peaks of 41 or 38 mL kg^−1^h^−1^ appeared at day 6 or 8 for naturally ripened fruit, without or with CST, respectively (Fig 9A), while the ethylene peak of 40.5 or 36 mL kg^−1^h^−1^ appeared at day 3 via exogenous ethylene ripening without or with CST, respectively (Fig 9B). Similar results were observed in tomato fruit upon CST due to the inhibition of ACC oxidase and ACC synthase activities, which might account for inhibited carbohydrate metabolism of apricots and sweet cherry fruit [26, 33]. Activities of PG, PME, and cellulose in association to firmness in avocado fruit were directly correlated with ripening processes such as climacteric rise of respiration and ethylene production rates [18, 34–35]. The less peel discoloration and pulp firmness loss of the fruit in response to CST in our study may imply that these qualities of “Hass” avocado may be partially, or even strictly, dependent on ethylene during ripening. However, more studies should be conducted in the future. The strict dependence of pear fruit softening or partial dependence of skin degreening and flesh softening in mountain papayas on ethylene during their ripening were reported by Hiwasa et al. (2003) [36] and Moya-Leon et al. (2004) [37], respectively.

## Conclusion

In this study, ice water immersion for 30 min effectively prolonged the shelf life of naturally ripened and ethylene-ripened ‘Hass’ avocado fruits. CST inhibited peel discoloration and firmness loss, suppressed PG and endo-β-1,4-glucanase activities, and reduced ethylene production rate and respiration rate. As a simple, safe, and inexpensive postharvest technology, utilizing CST to manipulate physiological and biochemical activities could be a practical approach to extend the green life and shelf life of avocado fruits during storage.

## Supporting information

S1 File Data of Figs 1, 3, 4, 5, 6, 7, 8 and 9 in S1 File. (DOC)
