# Supplementary material for: Cold shock treatment extends shelf life of naturally ripened or ethylene-ripened avocado fruits
Source: PLoS One. 2017 Dec 18;12(12):e0189991. doi: 10.1371/journal.pone.0189991 (PMC5734781; doi:10.1371/journal.pone.0189991)
Supplement: S1 File — (DOC) [file pone.0189991.s001.doc]

**S1 Table. Data of figure 1.** Changes in core temperature of avocado fruits treated with cold shock at 0, 2 or 4°C for 0 to 120 minutes (A) and the corresponding firmness after 6-day storage at 20°C and 85−90% RH (B). Different letters at any storage time indicate significant differences (P<0.05).

| **Duration**  **(minutes)** | **(A)** | | | | **(B)** | | | | |
| --- | --- | --- | --- | --- | --- | --- | --- | --- | --- |
|  | 0℃ | 2℃ | 4℃ | | 0℃ | 2℃ | | 4℃ | |
| 0  15  30  45  60  90  120 | 20±1.21a  9±0.72b  6±0.54b  5.8±0.62b  2.5±0.31b  2.2±0.32b  2±0.32b | 20±1.21a  11.5±0.81a  7.5±0.55a  7±0.52ab  5.5±0.54a  5±0.53a  4±0.21a | | 20±1.21a  12.5±0.79a  8±0.49a  7.5±0.48a  6±0.54a  5.3±0.53a  4±0.22a | 18±1.81a  55.8±2.32a  68.6±3.33a  25.21±1.51a  16.42±1.72a  16.52±2.20a  13.5±1.81a | | 18±1.81a  58.2±2.81a  45±2.74b  14.94±1.35b  15.73±1.83a  15.02±1.54a  17.21±1.85a | | 18±1.81a  56.58±2.90a  35±2.10c  16.8±1.66b  11.54±1.24b  16.18±1.78a  15.68±22a |

**S2 Table. Data of figure 3.** Skin color (lightness, chroma value, and hue angle) of avocado fruits upon natural (A) ripening or ethylene-induced ripening (B) with or without cold shock treatment (CST) during storage. Different letters at any storage time indicate significant differences (P<0.05).

|  | **Storage time (d)** | **Lightness** | | **Chroma** | | **Hue** | |
| --- | --- | --- | --- | --- | --- | --- | --- |
|  |  | Control | CST | Control | CST | Control | CST |
| **(A)** | 0  2  4  6  8  10 | 42.17±1.02a  35.15±1.61b  32.15±1.73b  30.15±1.71b  29.35±1.60a  27.3±1.54a | 42.17±1.02a  39±1.74a  37±1.65a  35±1.80a  30.15±1.72a  29.35±1.61a | 22.13±1.02a  17.56±0.89b  12.56±0.88b  9.56±0.71b  7.64±0.56b  5.35±0.5b | 22.13±1.02a  20±0.93a  17±0.77a  12±0.65a  9.56±0.52a  7.64±0.45a | 126.03±5.5 1a  120.85±5.02a  112.85±5.14a  95.85 ±4.78 b  72.67±4.24b  35.72 ±3.23b | 126.03±5.5 1a  122±5.12a  115±5.31a  110±5.22a  95.85 ±3.87a  72.67 ±3.42a |
| **(B)** | 0  1  2  3  4  5 | 42.17±1.02a  37.15±0.99a  34.15±1.05a  30.15±0.89b  28.35±0.88b  27.3±0.79b | 42.17±1.02a  39±1.03a  36±1.05a  34±0.97a  31.15±0.91a  29.35±0.89a | 22.13±1.02a  18.56±0.89a  13.56±0.88b  9.56±0.71b  7.64±0.56b  5.35±0.53b | 22.13±1.02a  20±0.91a  17±0.77a  13±0.65a  11±0.52a  7.64±0.45a | 126.03±5.5 1a  122.85±5.13a  112.85±5.41a  95.85±4.78b  72.67±4.22b  35.72±3.17b | 126.03±5.5 1a  122±5.02a  115±5.32a  110±5.05a  95.85±3.99a  72.67 ±3.88a |

**S3 Table. Data of figure 4.** Firmness of avocado fruits upon natural (A) or ethylene-induced (B) ripening with or without cold shock treatment (CST) during storage. Different letters at any storage time indicate significant differences (P<0.05).

|  | **Storage time (d)** | **Firmness (N)** | |
| --- | --- | --- | --- |
|  |  | Control | CST |
| **(A)** | 0  2  4  6  8  10 | 112±4.53a  95±3.02a  75±3.11b  35±1.92b  16±1.85b  8±1.8 1b | 112±4.53a  99±3.16a  90±3.24a  68±2.55a  32±2.20a  20±1.88a |
| **(B)** | 0  1  2  3  4  5 | 112±4.53a  88±3.23a  68±3.13b  38±1.96b  16±1.83b  4±1.8 0b | 112±4.53a  95±3.54a  80±3.33a  66±2.98a  39±2.21a  15±2.11a |

**S4 Table. Data of figure 5.** PG activity of avocado fruits upon natural (A) or ethylene-induced (B) ripening with or without cold shock treatment (CST) during storage. Different letters at any storage time indicate significant differences (P<0.05).

|  | **Storage time (d)** | **PG activity (μg.h^-1^g^-1^)** | |
| --- | --- | --- | --- |
|  |  | Control | CST |
| **(A)** | 0  2  4  6  8  10 | 110.21±20.42a  238.37±22.14a  325.52±28.12a  598.83±29.62a  656±30.21a  739.78±31.23a | 110.21±20.42a  156.59±18.74b  225.85±22.01b  375.85±21.76b  567±28.22b  675.87±25.51b |
| **(B)** | 0  1  2  3  4  5 | 110.21±20.42a  351.37±22.14a  501.52±28.12a  628.83±29.62a  741±30.22a  814±31.23a | 110.21±20.42a  274±18.74b  421.85±22.01b  511.85±28.12b  637±29.21b  705.87±31.02b |

**S5 Table. Data of figure 6.** PME activity of avocado fruits upon natural (A) or ethylene-induced (B) ripening with or without cold shock treatment (CST) during storage. Different letters at any storage time indicate significant differences (P<0.05).

|  | **Storage time (d)** | **PME activity (Units/μmol.min^-1^)** | |
| --- | --- | --- | --- |
|  |  | Control | CST |
| **(A)** | 0  2  4  6  8  10 | 360.12±20.11a  310.24±16.77a  240.16±15.34a  146.48±13.54a  100.13±10.24a  85.33±12.11a | 360.12±20.11a  280±15.34a  230.41±13.25a  166.23±10.11a  95.41±9.65a  66.61±9.67a |
| **(B)** | 0  1  2  3  4  5 | 360.12±20.11a  310.11±16.77a  245.23±15.34a  152.21±13.54a  100.31±11.99a  71.21±12.11a | 360.12±20.11a  300.01±15.34a  210.12±13.25b  145.11±10.11a  95.03±12.01a  66.31±9.67a |

**S6 Table. Data of figure 7.** Endo-β-1,4-glucanase activity of avocado fruits upon natural (A) or ethylene-induced (B) ripening with or without cold shock treatment (CST) during storage. Different letters at any storage time indicate significant differences (P<0.05).

|  | **Storage time (d)** | **Endo-β-1,4-glucanase (μg.h^-1^g^-1^)** | |
| --- | --- | --- | --- |
|  |  | Control | CST |
| **(A)** | 0  2  4  6  8  10 | 121.09±10.42a  394.53±42.14a  616.42±48.12a  815.47±49.62a  989±48.56a  1165.78±51.23a | 121.09±10.42a  278.60±28.74b  440.74±39.01b  554.61±41.76b  745±46.89b  894.88±45.51b |
| **(B)** | 0  1  2  3  4  5 | 121.09±10.42a  414.53±42.14a  596.42±48.12a  855.47±49.62a  1004.02±50.21a  1265.78±51.23a | 121.09±10.42a  322.61±28.74b  450.74±39.01b  614.61±41.76b  845±48.85b  984.88±45.51b |

**S7 Table. Data of figure 8.** Respiration rate of avocado fruits upon natural (A) or ethylene-induced (B) ripening with or without cold shock treatment (CST) during storage. Different letters at any storage time indicate significant differences (P<0.05).

|  | **Storage time (d)** | **CO2(mg/kg.h)** | |
| --- | --- | --- | --- |
|  |  | Control | CST |
| **(A)** | 0  2  4  6  8  10 | 40±1.42a  42±2.12a  53±1.23a  81±2.14a  70±1.62b  61±1.23b | 40±1.42a  41±1.74a  48±1.53b  57±1.76b  78±2.01a  65±1.51a |
| **(B)** | 0  1  2  3  4  5 | 40±1.42a  47±2.12a  63±1.23a  85±2.14a  74±1.62a  58±1.23a | 40±1.42a  44±1.74a  53±1.88b  79±2.01b  69±1.51b  61±1.76a |

**S8 Table. Data of figure 9.** Ethylene production rate of avocado fruits upon natural (A) or ethylene-induced (B) ripening with or without cold shock treatment (CST) during storage. Different letters at any storage time indicate significant differences (P<0.05).

|  | **Storage time (d)** | **C2H4 (μl/kg.h)** | |
| --- | --- | --- | --- |
|  |  | Control | CST |
| **(A)** | 0  2  4  6  8  10 | 0.1±0.02a  1±0.12a  3±0.72a  42±1.84a  5±0.82b  0.2±0.83b | 0.1±0.02a  0.1±0.12a  2±0.71a  6±1.14b  38±1.62a  10±0.63a |
| **(B)** | 0  1  2  3  4  5 | 0.1±0.02a  5±0.92a  15±1.11a  43±1.84a  20±0.82a  3±0.83a | 0.1±0.02a  3±0.62a  9±0.99b  36±1.68b  15±1.14b  4±0.63a |
